# Case-based reasoning for emergency response planning of coal mine gas explosion accidents

**DOI:** 10.1371/journal.pone.0331711

**Published:** 2025-09-11

**Authors:** Dongling Ma, Jingwen Kuang, Minyuan Zhang

**Affiliations:** 1 School of Management, Wuhan Technology and Business University, Wuhan, China; 2 School of Accounting, Wuhan Qingchuan University, Wuhan, China; 3 Business School, Macau University of Science and Technology, Macau, China; Henan Polytechnic University, CHINA

## Abstract

Gas explosions in coal mines pose a serious threat to miner safety and operational sustainability, often resulting in significant casualties and production losses. To address the deficiencies in emergency decision-making and preparedness, this study proposes a case-based reasoning (CBR) model for emergency response planning, using a representative gas explosion incident at Mine B as the target case. Historical accident cases were analyzed to extract and quantify key descriptive and decision-related attributes. A cloud model-based weighting method was employed to determine the relative importance of features, followed by improved K-nearest neighbor (KNN) retrieval for similar case matching. A multi-population genetic algorithm (MEA) was used to optimize the weights and thresholds of a backpropagation (BP) neural network for case adaptation and reuse. The cloud model was further introduced to evaluate the effectiveness of the proposed emergency plans. Simulation results demonstrate that the model yields reliable and practical emergency responses, with the evaluated plan rated between “fair” and “good.” Finally, this study outlines implementation and safeguard measures for emergency plan execution, offering a scientifically grounded reference for coal mine enterprises to enhance gas explosion preparedness and response efficiency.

## 1. Introduction

Harmful gases such as methane and explosive fumes pose significant threats to miners’ safety and the secure operation of coal mines. Among these, methane control is crucial for preventing major coal mine disasters [1]. From 2013 to 2023, gas-related accidents accounted for 51% of all major coal mine incidents in China, while the combined occurrences and fatalities caused by gas explosions, coal dust explosions, water inrushes, fires, and roof collapses reached 87% and 86%, respectively [2]. Gas explosions are inherently complex and dynamic, involving multiple hazardous factors [3], often resulting in severe casualties. In some cases, secondary or multiple gas explosions exacerbate the disaster, leading to even greater destruction. Effective emergency response measures are essential to minimizing accident losses [4] and mitigating the risks of secondary explosions.

In recent years, a growing number of scholars have conducted extensive research aimed at enhancing coal mine safety, focusing primarily on three domains: the construction of risk early-warning models [5–12], the development of safety monitoring systems [13–19], and the intelligent formulation of emergency response plans [20–28].

Risk early-warning models are designed to predict the likelihood and severity of gas explosion accidents. For instance, Niu et al. [5] proposed a behavior-based risk assessment method by integrating the HFACS-GE classification system with Bayesian networks to evaluate unsafe human actions in gas explosion scenarios. Li et al. [6] identified key risk factors contributing to gas explosions and developed a causal reasoning model using Bayesian networks. Additionally, Li et al. [7] introduced a dynamic multi-source early-warning approach that integrates ARIMA and the Transferable Belief Model (TBM). Meng et al. [8] further investigated the impact of unsafe behavior in underground coal mines and established a corresponding risk assessment framework. While these models effectively mine latent patterns from historical data and improve risk perception, they still face common challenges, including poor data quality, sample imbalance, and limited generalizability.

At the system and hardware level, researchers have leveraged the Internet of Things (IoT) and smart sensing technologies to enable real-time monitoring of the coal mine environment. Pudke et al. [17] developed a wireless coal mine monitoring system based on ZigBee and GSM technologies, integrated with LabVIEW and microcontroller platforms. Dey et al. [16] further designed a hazard monitoring system that combines IoT with a CNN-LSTM deep learning architecture, enabling the extraction of spatiotemporal features from sensor data to predict disaster risks more effectively. Although these systems enhance the detection of potential hazards, their operational stability remains limited by network latency, power consumption, and the harsh underground environment.

Recent studies have incorporated artificial intelligence theories and related technologies into the formulation of emergency response plans for coal mine gas explosion accidents. By analyzing and processing real-time emergency information, these approaches predict accident evolution, allowing for the development and evaluation of corresponding response plans, thereby outperforming traditional emergency decision-making methods in addressing coal mine gas explosion incidents. Tong et al. [20] combined Bayesian networks with the Delphi method to dynamically assess gas explosion accidents, providing a more accurate evaluation framework for emergency decision-making. To support theoretical advancements in coal mine emergency rescue planning, Wu et al. [21] investigated the attenuation characteristics of blast shock waves and the dispersion of hazardous gases, aiming to reduce unnecessary losses caused by uncoordinated rescue efforts. Furthermore, Zhao et al. [22] addressed the limitations of traditional coal mine emergency plans and telephone-based dispatch systems by optimizing emergency management mechanisms and resource allocation models. They proposed an integrated architecture and functional modules for a coal mine emergency rescue system, enabling intelligent accident response, rescue operations, and emergency management. To support evidence-based decision-making in prioritizing key safety improvement factors, Jiskani et al. [23] developed a mine safety assessment index and proposed an integrated evaluation model based on entropy weighting and grey clustering, tailored to Pakistan’s current mine safety conditions and future improvements. With advancements in machine learning and AI, Li et al. [24] proposed an emergency decision support method combining Generalized Regression Neural Networks (GRNN) and Computational Fluid Dynamics (CFD) modeling, allowing for the estimation of rescue personnel exposure to explosion risks, thereby improving the quality of rescue decisions.

Among various intelligent methods, case-based reasoning (CBR) has gained increasing attention due to its reliance on past experience and knowledge, making it particularly suitable for unstructured, experience-driven emergency decision-making. Liao et al. [29] developed a CBR-based system for generating emergency response plans for environmental pollution accidents. Amailef et al. [30] created an ontology-driven Mobile Emergency Response System (MERS) that integrates a CBR mechanism to enhance intelligent emergency response capabilities. Zhang et al. [31] through the analysis of coal mine gas explosion accident reports, identified 28 typical risk factors and 16 coupling factors, and constructed an eight-level risk coupling structure using the ISM-NK model. Collectively, these studies demonstrate that CBR not only enables the reuse of successful past cases, but also supports the adaptation and optimization of emergency plans for specific scenarios, underscoring its high practical value.

With the rapid advancement of artificial intelligence and information technologies, an increasing number of disciplines are shifting towards intelligent solutions, including the development of emergency response plans. While existing studies have laid a solid theoretical and technical foundation, research on emergency planning for specific high-risk scenarios remains insufficient. Coal mine gas explosions, characterized by high uncertainty and complex evolution, present unique challenges for emergency response. Although CBR has shown initial promise in generating emergency plans for such accidents, current approaches still suffer from limitations in case representation, retrieval accuracy, and reuse strategies. Moreover, there is a notable lack of systematic research on gas explosion scenarios: historical accident data remain underutilized, key contributing factors and characteristic patterns are rarely explored, and the effectiveness of existing plans is seldom evaluated. Most prior work focuses on general coal mine emergencies, with little attention paid to the unique demands of gas explosion incidents. Addressing these gaps, this study proposes a novel CBR-based framework tailored to the specific attributes of gas explosion accidents. By integrating case representation, retrieval, reuse, and implementation evaluation into a unified approach, our work not only advances the methodological frontier but also offers practical value for data-driven, scenario-specific emergency response planning in high-risk industrial environments.

To address the challenges in the emergency response planning for gas explosion incidents in Mine B, this study adopts a CBR-based approach to identify the most relevant historical emergency plans, which are then adapted and optimized for reuse. The objective is to minimize the impact of gas explosion accidents, ensure the successful execution of emergency rescue operations, and provide a practical reference for the rapid and effective development of emergency response plans in coal mines.

## 2. Current status and challenges in the emergency response planning for gas explosion accidents in mine B

### 2.1 Current status of emergency response planning for gas explosion accidents in mine B

**Fig 1:**
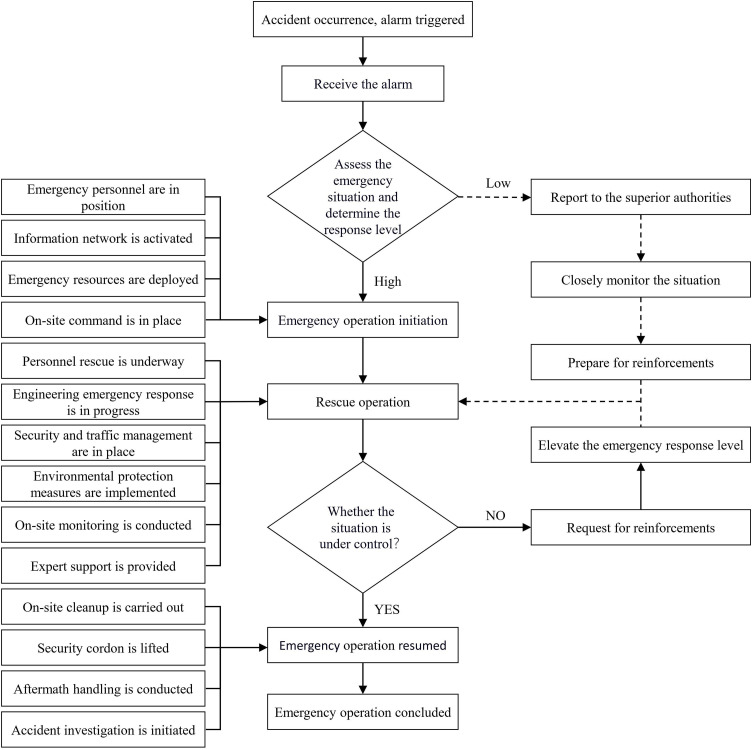
Emergency response procedure for coal mine B.

The emergency response system of Mine B primarily consists of an emergency command center, an on-site emergency rescue command unit, and an emergency rescue team. The formulation of the emergency response plan for gas explosions mainly includes prevention and early warning, emergency response, and information dissemination. The detailed emergency response procedures for gas explosion incidents are illustrated in Fig 1.

**Fig 2:**
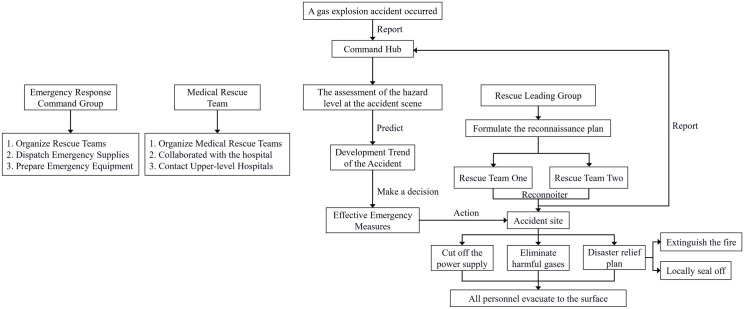
On-site emergency response process for gas explosion accident at coal mine B.

During accident handling, it is essential to determine the location, nature, and severity of the incident, as well as the surrounding gas conditions. The power supply must be cut off, hazardous gases must be eliminated to prevent further escalation, and any resulting fires must be extinguished. If the fire cannot be controlled, localized sealing measures should be implemented. Additionally, support structures should be restored to reestablish a stable production system. The detailed process is illustrated in Fig 2. The emergency response procedures and on-site handling processes for coal mine accidents depicted in Figs. 1 and 2 are designed based on the specific geological conditions and gas hazard levels of Mine B. Therefore, the model framework and related protocols reflect the actual operational environment and emergency response characteristics of Mine B.

### 2.2 Implementation results of the emergency response plan for gas explosion accidents in mine B

Following the established emergency response procedures, the timeline from the occurrence of the accident to the completion of the emergency response was analyzed to identify issues in the execution of Mine B’s gas explosion emergency response plan. On November 18 at 06:00, the high-grade general mining team was divided into two groups: a mechanized mining group and a drilling and blasting group, working at the coal pillar recovery face and the 9102 high-grade general mining face, respectively. At 13:07, workers in the drilling and blasting group violated operational regulations by conducting an unauthorized blast, which generated an open flame. This ignited methane that had infiltrated from the goaf of the 9103 working face into the coal pillar recovery area, triggering a gas explosion. At 13:50, the shift leader reported the accident to the dispatch center via telephone. At 14:31, the chief engineer informed the mine director by phone, who then reported the incident to the emergency management department.

Following the gas explosion, Mine B promptly initiated emergency response measures. The specific handling details are summarized in Table 1.

**Table 1:** On-site emergency response in mine B.

Time	Response actions
**13:56**	At the time of the accident, 105 workers were on duty, with 35 in the affected area. A total of 81 workers self-evacuated, while 24 were trapped. Upon receiving the accident report, the chief engineer notified all mine executives and department personnel to assemble at the dispatch center, initiated the emergency response plan, and organized rescue efforts, successfully saving five injured workers.
**16:10**	The national mine emergency rescue team (Fenxi team) received deployment orders for the accident response.
**16:12**	The leadership of the National Mine Rescue Center led four squads from the second and fourth rescue teams, dispatching 52 rescuers and nine rescue vehicles to the accident site.
**17:29**	The mine rescue brigade arrived at the accident site and proceeded to the command center for a situational briefing.
**17:52**	A detailed rescue plan was formulated, and a team of 19 rescuers was deployed underground to conduct reconnaissance and rescue trapped personnel.
**21:55**	Four trapped miners were rescued, and nine deceased workers were recovered. The command center ordered all rescuers to return to the surface.
**22:35**	A team of nine rescuers, together with the mine rescue squad, re-entered the mine to retrieve the remaining six deceased workers.
**02:30** **(Next Day)**	The final six deceased miners were brought to the surface. All rescuers exited the mine, marking the completion of the rescue operation.

Investigations revealed that, for over a month prior to the accident, Mine B had been engaging in unauthorized coal pillar recovery operations. During this period, three safety inspections were conducted, and each shift was overseen by a mine leader and safety inspectors. However, serious violations and major accident hazards were repeatedly overlooked.

Following the explosion, Mine B delayed reporting the incident for 40 minutes, exceeding the 30-minute regulatory requirement, classifying it as a late report. Additionally, the on-site emergency response was overly dependent on external rescue teams, with no immediate reconnaissance efforts initiated. The higher-level rescue teams only received deployment orders more than two hours after the explosion, resulting in delayed rescue operations.

These critical shortcomings significantly contributed to severe casualties and substantial economic losses.

### 2.3 Existing issues in the emergency response planning for gas explosion accidents in mine B

Mine B formulated its emergency rescue plan based on legal and regulatory guidelines. However, it lacks tailored protocols that reflect the mine’s specific operational conditions. The decision-making process remains fragmented, and the response measures and plan descriptions fail to form a coherent system. The emergency plan is largely formalistic, misaligned with actual on-site emergency handling, and suffers from delayed response times. Additionally, there are no reference emergency plans based on similar past incidents.

During on-site emergency handling, decision-makers exhibited overconfidence, evaded responsibility, and took excessive time to make critical decisions. The implemented measures were insufficiently targeted, lacking reference emergency rescue strategies. Furthermore, inadequate training on emergency plans resulted in poor execution.

Given these shortcomings, CBR technology can be employed to enhance gas explosion emergency planning. By leveraging extensive rescue experience, this approach can assist decision-makers, improve response efficiency, and enhance the overall effectiveness of mine rescue operations.

## 3. Construction of a case-based reasoning model for gas explosion emergency response planning

### 3.1 Case-based reasoning work model

**Fig 3:**
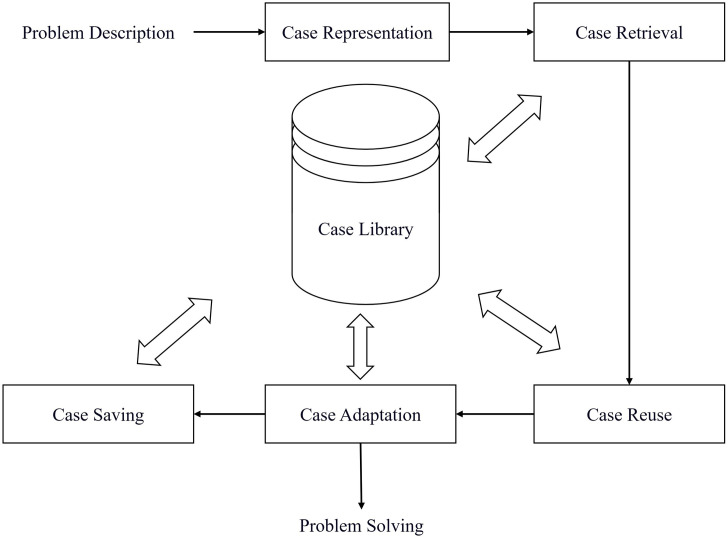
CBR problem-solving process.

This study adopts CBR technology, which consists of five key steps: case representation, retrieval, reuse, adaptation, and storage. By integrating historical solutions into a case database, decision-makers can access a broader set of references, improving emergency response planning. The overall workflow is illustrated in Fig 3.

To enhance the effectiveness of emergency response plan implementation in Mine B, a CBR model for gas explosion emergency response planning is constructed. This model consists of four main components: accident case representation, retrieval of similar cases, case reuse, and emergency plan evaluation.

### 3.2 Characteristic attributes and case representation of gas explosion accidents in coal mines

#### 3.2.1 Characteristic attributes of gas explosion accidents in coal mines.

(1) **Causal analysis of coal mine gas explosion accidents**

Cases serve as concrete manifestations and carriers of knowledge, requiring a comprehensive understanding of their characteristics and attributes. First, an analysis of accident causation enhances the relevance of emergency planning [32]. A structured and standardized description of accident cases is achieved by establishing an accident attribute system based on representative characteristic indicators. To conduct a more comprehensive analysis of the characteristics of coal mine gas explosion accidents, Python was used to crawl over 300 gas explosion accident cases from the China Coal Mine Safety Production website, leveraging third-party libraries such as urllib, xpath, and openpyxl. All data utilized in this study were sourced from the official China Coal Mine Safety website. Data collection and analysis were conducted in strict accordance with the website’s terms and conditions, ensuring full legal compliance without infringing upon any third-party rights. The collected accident case data, supplemented by relevant literature [33], were analyzed in terms of four key aspects: human factors, machinery and equipment, environmental factors, and management factors.

In coal mining operations, improper task allocation poses significant safety risks. For instance, assigning unlicensed personnel to blasting operations and insufficient safety education and skill training [34] can greatly increase the likelihood of accidents. Additionally, workers’ mental and physical conditions may impact operational safety, potentially leading to incidents. Regulatory negligence, such as ineffective supervision, disregard for existing hazards, and a lack of enforcement in personnel monitoring systems, further exacerbates risks [34].

Regarding mechanical factors, equipment failure rates tend to be higher during both early and late operational stages, and interactions between different machinery components may trigger cascading failures. The random nature of mechanical failures in coal mining operations poses a significant challenge, as certain equipment malfunctions may lead to ignition sources and excessive gas concentrations.

Environmental factors, including gas concentration, airflow velocity, and power supply stability, also influence accident risks. Gas concentration levels are affected by spatial distribution, geological conditions, and mining intensity, while airflow velocity is determined by ventilation systems, air doors, and air windows.

Unlike human, mechanical, and environmental factors, management deficiencies serve as indirect causes of accidents. Organizational structure, training programs, and assessment mechanisms play a crucial role in accident prevention. Ultimately, human, mechanical, and environmental risks often stem from underlying management flaws that need to be addressed for comprehensive safety improvement.

(2) **Extraction of fundamental attributes of accidents**

Coal mine gas explosions are typically caused by multiple factors, including human, mechanical, environmental, and managerial elements [35]. However, organizational and management factors are often underrepresented in existing analyses [36]. Based on statistical analyses of past coal mine gas explosion accidents, this study examines four key aspects: fundamental accident information, explosion location, causes of gas explosions, and safety management practices. To minimize retrieval workload and enhance efficiency, attributes with negligible impact or difficult accessibility are excluded, resulting in the selection of 18 fundamental attributes. The raw attribute values are standardized, and the accident severity is considered by assigning a “1～4” characteristic value to each attribute, where higher values indicate a greater probability of severe accidents. The classification criteria are detailed in Table 2.

**Table 2:** Characteristic values of fundamental accident attributes.

Fundamental Accident Attributes		Characteristic Values			
		1	2	3	4
Fundamental Accident Information	Accident Time	00:00–06:00	06:00–12:00	12:00–18:00	18:00–24:00
	Nature of Accident	Responsible Accident	—	—	Non-Responsible Accident
	Severity Level	Minor Accident	Relatively Major Accident	Major Accident	Particularly Severe Accident
	Economic Loss (thousand CNY)	<1000	1000–5000	5000–10000	≥10000
	Number of Trapped Individuals	0–10	10–30	30–50	>50
	Number of Fatalities	0–3	3–10	10–30	>30
	Number of Injuries	0–3	3–10	10–30	>30
	Number of Missing Individuals	0–3	3–10	10–30	>30
Gas Explosion Zone	Secondary or Multiple Explosions	Low	High	Very High	Extremely High
	Fire Impact Scope	Small	Moderate	Large	Extremely Large
	Explosion Impact Area	Small	Moderate	Large	Extremely Large
	Explosion Location [37]	Coal Mining Face	Tunneling Face	Goaf	Roadway
Causes of Gas Explosion	Ventilation System Status	Good	Moderate	Damaged	Severely Damaged
	Illegal Operations	Few	Moderate	Many	Extremely Many
	Electromechanical Equipment Factors	Minor	Moderate	Major	Extremely Major
	Organizational and Management Factors	Minor	Moderate	Major	Extremely Major
Safety Management	Employee Training Status	—	Moderate	Poor	Extremely Poor
	Safety Management Status	Good	Moderate	Poor	Extremely Poor

(3) **Extraction of decision attributes for emergency response plans**

Following an accident, an emergency response plan serves as a crucial decision-support tool. In scenarios where incidents are sudden, rapidly evolving, and highly destructive [24], relying solely on decision-makers’ intuition is insufficient. Instead, emergency response plans provide pre-established frameworks for emergency rescue operations, formulated through in-depth analysis of accident progression mechanisms. Although these plans cannot fully align with real-time on-site conditions, they offer structured guidance for rescue efforts.

Emergency response plans specify essential organizational and individual roles, necessary resources, equipment guarantees, and predefined rescue capabilities. By learning from similar past cases, emergency response plans can be refined and improved to enhance preparedness.

Through an extensive investigation of emergency response procedures across numerous accident cases, the emergency response process is primarily divided into three key stages: emergency activation, rescue operations, and post-emergency recovery. The specific methodologies associated with these stages are outlined in Table 3.

**Table 3:** Characteristic values of decision attributes in emergency response plans.

Decision attribute	Characteristic values	
	1	0
Emergency Rescue Command Center	Adopted	Not Adopte
Comprehensive Coordination	Adopted	Not Adopte
Establishment of Underground Rescue Base	Adopted	Not Adopte
On-Site Monitoring	Adopted	Not Adopte
Disaster Area Reconnaissance	Adopted	Not Adopte
Ventilation Restoration	Adopted	Not Adopte
Direct Firefighting	Adopted	Not Adopte
Sealed Firefighting	Adopted	Not Adopte
Water Injection Firefighting	Adopted	Not Adopte
Engineering Emergency Operations	Adopted	Not Adopte
Transporting Deceased Victims	Adopted	Not Adopte
Rescue of Trapped Individuals	Adopted	Not Adopte
On-Site Cleanup	Adopted	Not Adopte
Medical Treatment	Adopted	Not Adopte
Public Opinion Guidance	Adopted	Not Adopte
Logistics Support	Adopted	Not Adopte
Post-Accident Aftercare	Adopted	Not Adopte

The decision-making framework for emergency response plans consists of 17 attributes, each assigned a characteristic value of 1 or 0 (1 indicates the method is incorporated into the emergency plan, while 0 signifies its absence).

(4) **Extraction of evaluation indicators for emergency response plans**

Simple casualty counts and economic losses are insufficient to assess the effectiveness of emergency plans. Instead, evaluation should be conducted by linking implementation outcomes to the formulation of emergency plans. Based on previous assessment indicators for emergency plans in past incidents, this study further considers emergency response procedures and historical accident cases. The effectiveness of plan implementation is evaluated from three perspectives: completeness, operability, and rationality. Each indicator is standardized and classified into five levels, ranging from excellent to poor, with characteristic values presented in Table 4.

**Table 4:** Characteristic values of emergency plan evaluation indicators.

Evaluation indicator	Characteristic values				
	90-100	80-90	70-80	60-70	50-60
Hazard Identification	Excellent	Good	Average	Poor	Very Poor
Responsibilities of Rescue Agencies	Excellent	Good	Average	Poor	Very Poor
Post-incident Disposal	Excellent	Good	Average	Poor	Very Poor
On-site Response	Excellent	Good	Average	Poor	Very Poor
Emergency Tasks	Excellent	Good	Average	Poor	Very Poor
Disposal Procedures	Excellent	Good	Average	Poor	Very Poor
Information Dissemination	Excellent	Good	Average	Poor	Very Poor
Timeliness of Early Warning	Excellent	Good	Average	Poor	Very Poor
Execution Order	Excellent	Good	Average	Poor	Very Poor
Safeguard Measures	Excellent	Good	Average	Poor	Very Poor
Incident Containment Capability	Excellent	Good	Average	Poor	Very Poor

#### 3.2.2 Representation of coal mine gas explosion accident cases.

By analyzing the attributes of accident characteristics and addressing practical problem-solving needs, coal mine gas explosion accident cases are represented in the form of a triplet, which serves as the foundation for constructing a case database. The case database consists of three components: accident problem description, solution, and implementation effectiveness. Specifically, the accident problem description captures basic characteristic attributes, the solution represents decision-making attributes, and the implementation effectiveness serves as an evaluation metric. The problem description facilitates the retrieval of similar cases, from which the corresponding solution and implementation effectiveness are extracted based on similarity calculations. Consequently, a case is represented as:


Case={Problem,Solution,Result}\]

where Case denotes an emergency case of a gas explosion accident, and the historical emergency case set is denoted as *C* = {*C*₁, *C*₂, *C*₃, …, *C*_T_}, forming the case database. Problem (*P*) represents the problem description and consists of m basic characteristic attributes, forming the emergency case problem attribute set *P* = {*P*₁, *P*₂, *P*₃, …, *P*ᵢ}. Solution (*S*) represents the emergency response strategy, forming the emergency plan set *S* = {*S*₁, *S*₂, *S*₃, …, *S*k}. Result (*R*) represents the implementation effectiveness, forming the implementation evaluation set *R* = {*R*₁, *R*₂, *R*₃, …, *R*ⱼ}. The target case, denoted as *C*₀, corresponds to the gas explosion accident at B Coal Mine, with its problem description represented as *P*₀. Here, T, i, j, and k are positive integers.

Accident case representation serves as the foundation and prerequisite for decision-makers to formulate effective plans, making it well-suited for a combination of frame-based and production rule representations. In this approach, accident characteristic attributes are embedded within a structured framework, defined as Frame Name = < Slot *P*: Problem, Slot *S*: Solution, Slot *R*: Result > . The Problem (*P*) slot encompasses facets such as *P*_1_: Accident Time, *P*_2_: Nature of Accident, …, *P*_18_: Safety Management Status. The Solution (*S*) slot includes elements like *S*_1_: Emergency Rescue Command Center, *S*_2_: Comprehensive Coordination, …, *S*_17_: Post-Accident Aftercare. Finally, the Result (*R*) slot captures key aspects such as *R*_1_: Hazard Identification, *R*_2_: Responsibilities of Rescue Agencies, …, *R*_11_: Incident Containment Capability.

### 3.3 Similar case retrieval based on the k-nearest neighbors (KNN) algorithm

#### 3.3.1 Determination of basic feature attribute weights.

**Fig 4:**
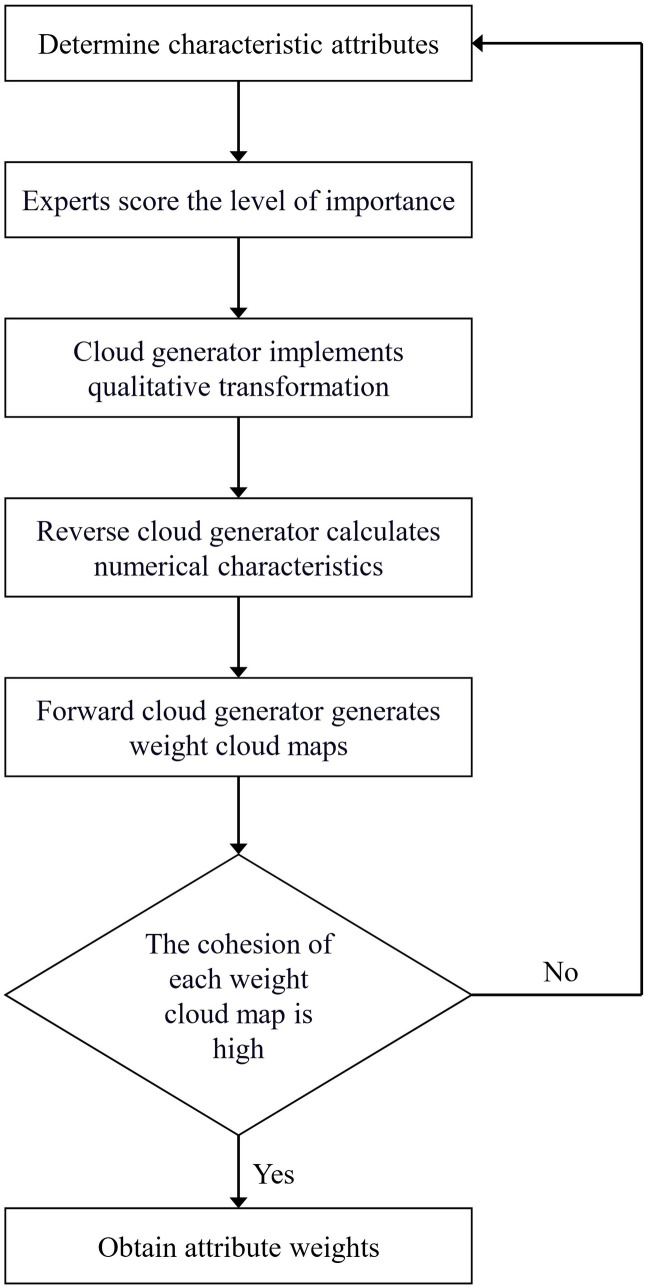
Workflow for determining the weights of case attributes.

Before conducting CBR retrieval, it is necessary to determine the weights of basic feature attributes due to their varying importance. A combined subjective-objective weighting method is employed, which not only considers the decision-maker’s subjective judgment but also reflects the objective relationships in the data. Since the problem descriptions of accident cases contain both quantitative and qualitative data, there may be instances of incomplete or uncertain data. To address this, this study integrates the expert scoring method with the cloud model to ensure the objectivity of attribute weighting using expert knowledge and experience. The specific weighting process is illustrated in Fig 4. The cloud model, based on probability theory and fuzzy set theory, serves as a transformation model for handling quantitative and qualitative uncertainty [38]. By constructing an uncertainty transformation model, it effectively represents the relationship between qualitative and quantitative attributes, thus resolving the challenge of weighting qualitative data.

**Fig 5:**
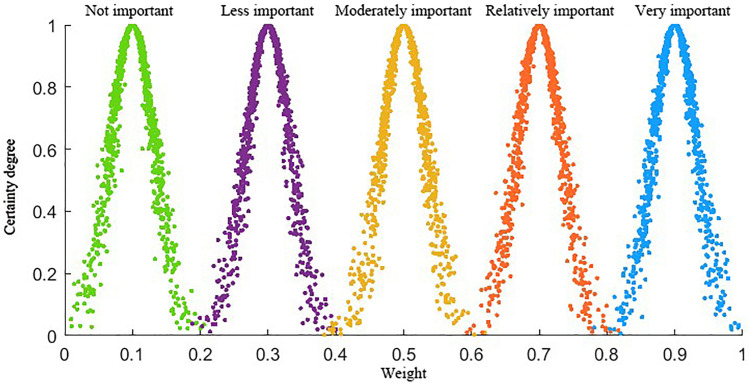
Feature attribute weight cloud map.

The numerical distribution range of attribute weights, qualitative descriptions, and cloud characteristic values for the case study are established, as shown in Table 5. The numerical characteristics of the cloud model are represented by expectation (*E*_*x*_), entropy (*E*_*n*_), and hyper-entropy (*H*_*e*_). The cloud map of basic attribute weights is presented in Fig 5.

**Table 5:** Numerical distribution range, qualitative descriptions, and cloud characteristic values of case attribute weights.

Weight range	[0.8~1.0]	[0.6 ~ 0.8)	[0.4 ~ 0.6)	[0.2 ~ 0.4)	[0.0 ~ 0.2)
Importance Level	Very Important	Relatively Important	Moderately Important	Less Important	Not Important
Expectation	0.9	0.7	0.5	0.3	0.1
(*E*_*x*_, *E*_*n*_, *H*_*e*_)	(0.9,0.033,0.005)	(0.7,0.033,0.005)	(0.5,0.033,0.005)	(0.3,0.033,0.005)	(0.1,0.0.033,0.005)

#### 3.3.2 Similar case retrieval.

Case retrieval is a crucial step in CBR [39]. To enhance retrieval efficiency and accuracy, an improved similarity retrieval method based on the commonly used KNN algorithm is proposed. In KNN, a case is represented as a feature vector *V*_*Pi*_, composed of multiple fundamental attribute features. Similarity retrieval is performed by calculating the similarity between cases through the distance *D*($V_{P_{i}}$, $V_{P_{0}}$), which determines the case similarity *sim*($V_{P_{i}}$, $V_{P_{0}}$). Generally, a smaller distance *D*($V_{P_{i}}$, $V_{P_{0}}$) corresponds to a higher similarity value *sim*($V_{P_{i}}$, $V_{P_{0}}$), indicating that the two cases are more alike. The similarity score is constrained within the range *sim*($V_{P_{i}}$, $V_{P_{0}}$)∈[0,1].

Given that different fundamental attributes have varying degrees of influence, a weighted Euclidean distance is employed to measure similarity, introducing weight coefficients to represent the relative importance of each attribute. The similarity between a target case and historical cases *sim*(_*Pj*_, _*P0*_) is calculated using the following formula:


$${sim}_{\left(V_{P_{i}}\text{,}V_{P_{0}}\right)}=1-\sqrt{\sum_{s=1}^{i}\;\omega_{P_{i}}D_{\left(V_{P_{is}},V_{P_{0s}}\right)}}$$ (1)

where the relative distance between the target case and historical cases is given by:


$$D_{\left(V_{P_{i}}\text{,}V_{P_{0}}\right)}=\frac{\left|V_{P_{is}}-V_{P_{0s}}\right|}{{max}_{s}-{min}_{s}}$$ (2)

In these equations, $V_{P_{is}}$ and $V_{P_{is}}$ represent the *s*-th fundamental attribute of the historical and target cases, respectively. The weight coefficient $\omega_{P_{i}}$ denotes the importance of each fundamental attribute in assessing the case similarity. *max*_*s*_ and *min*_*s*_ correspond to the maximum and minimum values of the *s*-th fundamental attribute.

### 3.4 Case reuse based on mind evolutionary algorithm (MEA) BP neural network

After retrieving similar cases, they often cannot be directly applied to the target case and require adaptation and modification to obtain the final solution. Traditionally, case reuse is conducted based on expert opinions and reference to the retrieved similar cases. However, this method is inherently subjective. To mitigate this, the MEA-BP neural network is introduced.

**Fig 6:**
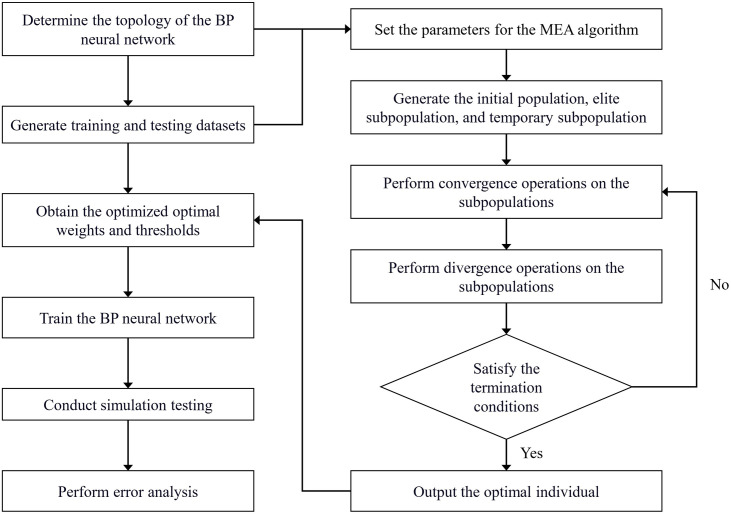
MEA-BP neural network algorithm flowchart.

The MEA simulates the human cognitive evolution process and incorporates the structural design principles of the Genetic Algorithm (GA). It enriches the concepts of “convergence” and “divergence” and exhibits a strong global search and optimization capability from a population perspective [40]. By leveraging MEA to optimize the weight and threshold parameters of the BP neural network, the MEA-BP neural network enhances case reuse. The specific workflow of the MEA-BP neural network algorithm is illustrated in Fig 6.

### 3.5 Emergency plan evaluation based on the cloud model

The cloud model is employed to assess the reused emergency plan using evaluation indicators. The evaluation values from *n* experts are input into the cloud model, and the inverse cloud generator is utilized to compute the characteristic value *R*_*j*_(*E*_*x*_, *E*_*n*_, *H*_*e*_), where *E*_*x*_ represents the cloud model evaluation value for each indicator. The evaluation criteria are divided into five levels (Excellent, Good, Average, Poor, and Very Poor, and Very Poor) corresponding to characteristic values of 95, 85, 75, 65, and 55, respectively. The specific implementation steps of the cloud model evaluation are as follows:

#### Step 1: Determining indicator weights and defining evaluation standards.

For the *j*-th evaluation indicator, its weight $\omega_{R_{j}}$ is determined, and the evaluation standard is defined. The numerical characteristics of the cloud are calculated as follows, where *O*_*min*_ and *O*_*max*_ represent the lower and upper bounds of the interval, and *K* is set to 0.005:


$$E_{x}=\left(O_{max}+O_{min}\right)/2$$ (3)

$$E_{n}=\left(O_{max}-O_{min}\right)/6$$ (4)

$$H_{e}=K$$ (5)

#### Step 2: Computing the comprehensive evaluation cloud.

Using the inverse cloud generator, the evaluation cloud *R*_*t*_(*E*_*xt*_, *E*_*nt*_, *H*_*et*_) for the *t*-th indicator is computed. The comprehensive evaluation cloud *N*(*E*_*x*_, *E*_*n*_, *H*_*e*_) of the emergency plan implementation is then determined as follows:


$$E_{x}=\sum_{t=1}^{j}\omega_{R_{jt}}E_{xjt}\;$$ (6)

$$E_{n}=\sqrt{\sum_{t=1}^{j}\omega_{R_{jt}}E_{njt}^{2}\;}$$ (7)

$$H_{e}=\sum_{t=1}^{j}\omega_{R_{jt}}H_{ejt}\;$$ (8)

#### Step 3: Determining the evaluation level.

Finally, MATLAB is used to compare the comprehensive evaluation cloud with the standard evaluation clouds. By analyzing the resulting cloud maps, the overall effectiveness level of the emergency plan is preliminarily determined.

## 4. Simulation and safeguard measures for the emergency response plan of gas explosion in B coal mine

### 4.1 Simulation of emergency response plan for gas explosion in B coal mine

This study takes the emergency response plan for gas explosion accidents in B Coal Mine as a case study. A simulation test is conducted to improve and refine the plan, verifying its feasibility and effectiveness.

#### 4.1.1 Establishing a case database for gas explosion accidents in coal mines.

Following the gas explosion accident in B Coal Mine, the incident was not reported within the required time frame. The complexity of accident information, along with decision-makers prioritizing economic interests over safety, led to delays in emergency response measures. The existing emergency plan of B Coal Mine is overly formalized, misaligned with actual on-site handling procedures, and lacks a thorough analysis of emergency response capabilities. Additionally, no adjustments were made to the emergency measures, resulting in poor effectiveness in the gas explosion rescue operation.

A dataset of 50 coal mine gas explosion incidents was curated from publicly available reports on the China Coal Mine Safety website, covering events since 2000. Only cases with complete records, well-defined attributes, and high comparability were retained following rigorous screening; duplicate entries and pre-2000 incidents were excluded. Although modest in size, the dataset reflects the scarcity and technical specificity of high-quality incident records, which justifies its use in this study Given these constraints, we employed a CBR framework in combination with a MEA-BP neural network, both of which are well-suited to small-sample settings. This approach prioritizes knowledge transfer and inference accuracy over reliance on large-scale training data. The target case (B Coal Mine gas explosion) and historical cases were systematically represented and standardized for analysis, as shown in Table 6.

**Table 6:** Characteristic values of basic attributes for gas explosion accident cases in coal mines.

Basic Attributes	*C*_0_	*C*_1_	*C*_2_	*C*_3_	*C*_4_	…	*C*_50_
*P*_1_	3	1	3	3	3	…	3
*P*_2_	1	1	1	1	1	…	1
*P*_3_	3	2	2	2	2	…	4
*P*_4_	2	2	1	1	1	…	1
…	…	…	…	…	…	…	…
*P*_18_	4	3	3	3	4	…	3

#### 4.1.2 Retrieval of similar cases.

(1) **Weight assignment for basic characteristic attributes**

To assign weights to the basic characteristic attribute accident occurrence time (*P*_1_), the expert scoring method was used. A total of eight experts were invited to participate in the evaluation. The first round of expert scores was recorded as:


$$x_{1}=[0.15,0.28,0.45,0.30,0.28,0.31,0.26,0.50]$$

Using the cloud generator, the expert scores were transformed from qualitative assessments into quantitative representations, yielding the cloud model characteristic values for accident occurrence time (*P*_1_):


$$N_{1}(E_{x},E_{n},H_{e})=(0.3163,0.0995,0.0480)$$

**Fig 7:**
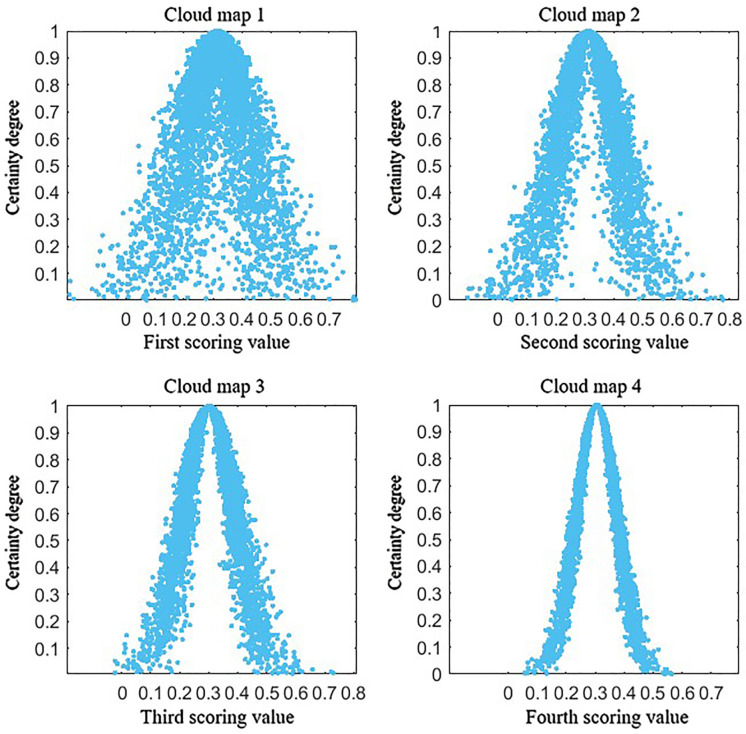
Time of incident (P1) attribute weights acquisition cloud map.

A forward cloud generator was then employed to generate the expert evaluation value cloud map for accident occurrence time (*P*_1_), as illustrated in Fig 7.

In Fig 7, cloud map 1 displays a highly dispersed cloud with a foggy appearance, indicating significant disagreement among experts in the first round of scoring. The results were compiled and fed back to the experts for reassessment, and the process was repeated until a clear aggregation pattern emerged.

Second round of scoring:


$$x_{2}=[0.17,0.26,0.43,0.33,0.26,0.29,0.27,0.49]$$

Cloud map 2 demonstrated a more clustered pattern compared to the first round, indicating improved consensus.

Third round of scoring:


$$x_{3}=[0.17,0.25,0.40,0.34,0.25,0.26,0.30,0.45]$$

Cloud map 3 showed further aggregation, signifying increasing agreement among experts.

Fourth round of scoring (final result):


$$x_{4}=[0.20,0.25,0.39,0.34,0.29,0.26,0.31,0.40]$$

Cloud map 4 displayed a distinct clustering pattern, indicating that expert opinions on the importance of accident occurrence time (*P*_1_) had reached a consensus.

From the final cloud map, the characteristic values for the fourth round were extracted as: *N*_4_(*E*_*x*_, *E*_*n*_, *H*_*e*_)=(0.3050, 0.0689, 0.0088) where *E*_*x*_ represents the weight value of accident occurrence time (*P*_1_). Qualitatively, accident occurrence time (*P*_1_) was classified as “moderately important”.

Following the scoring method and cloud model approach, the weight values for the basic characteristic attributes were determined, as summarized in Table 7.

**Table 7:** Weight values of basic characteristic attributes.

Basic attributes	Weight value	The weight values after normalization	Basic attributes	Weight value	The weight values after normalization
*P*_1_	0.3050	0.0389	*P*_10_	0.5364	0.0683
*P*_2_	0.0000	0.0000	*P*_11_	0.5426	0.0691
*P*_3_	0.6231	0.0794	*P*_12_	0.3164	0.0403
*P*_4_	0.2551	0.0325	*P*_13_	0.5395	0.0687
*P*_5_	0.6153	0.0784	*P*_14_	0.4126	0.0526
*P*_6_	0.6451	0.0822	*P*_15_	0.5424	0.0691
*P*_7_	0.5422	0.0691	*P*_16_	0.4264	0.0543
*P*_8_	0.2152	0.0274	*P*_17_	0.3425	0.0436
*P*_9_	0.6425	0.0819	*P*_18_	0.3468	0.0442

(2) **Similarity calculation**

Based on the weight values of the fundamental feature attributes described in the problem statement, the improved KNN algorithm is employed to compute the similarity between historical cases and the target case. The results are presented in Table 8.

**Table 8:** Computation results of similar cases.

Case ID	Accident Case	Similarity
10	*C*_10_	0.9036
9	*C*_9_	0.8476
17	*C*_17_	0.8041
7	*C*_7_	0.6953
35	*C*_35_	0.6602
8	*C*_8_	0.6501
14	*C*_14_	0.6469
4	*C*_4_	0.6264

Cases 10, 9, 17, 7, 35, 8, 14, and 4 rank among the top eight most similar cases to the target case. The emergency response decision attribute values of these highly similar cases are detailed in Table 9.

**Table 9:** Emergency response decision attribute values of similar cases.

Decision Attribute	*C*_0_	*C*_10_	*C*_9_	*C*_17_	*C*_7_	*C*_35_	*C*_8_	*C*_14_	*C*_4_
*S*_1_	TBD	1	1	1	1	1	1	1	1
*S*_2_	TBD	1	1	1	1	1	1	1	1
*S*_3_	TBD	0	1	0	0	1	0	0	0
*S*_4_	TBD	0	1	1	1	1	1	1	0
…	…	…	…	…	…	…	…	…	…
*S*_17_	TBD	1	1	1	1	1	1	1	1

#### 4.1.3 Reuse of emergency response plans from similar cases.

The retrieved eight similar cases are used as samples to predict the emergency response plan for the target case using the MEA-improved BP neural network. Attributes with identical feature values in decision attributes and basic feature attributes, specifically *S*_1_, *S*_2_, *S*_5_, *S*_13_, *S*_14_, *S*_16_, *S*_17_, *P*_2_ and *P*_8_, are removed. The prediction is conducted using the remaining 10 decision attributes and 16 basic feature attributes, where all identical decision attribute values are adopted.

For the BP neural network, the input layer consists of 16 nodes, while the output layer consists of 10 nodes. The number of hidden layer nodes is determined based on the empirical formula:


$$l<\sqrt{m+n}+\tau$$ (9)

where *l* represents the number of hidden layer nodes, *m* is the number of input layer nodes, *n* is the number of output layer nodes, and τ is an adjustment parameter typically ranging from 1 to 10. Experimental tests on different hidden layer sizes indicate that when the number of hidden layer nodes is set to 8, the mean squared error (MSE) of the network is minimized. The BP neural network training algorithm establishes the relationship between inputs and expected outputs using the newrff function, while MEA optimizes the initial weights and thresholds of the BP neural network. The parameter settings for the code are provided in Table 10.

**Table 10:** Parameter settings.

Parameter name	Value	Parameter name	Value
Input layer nodes	16	Population size	100
Output layer nodes	10	Elite subpopulation size	5
Hidden layer nodes	8	Temporary subpopulation size	5
Maximum training iterations	100	Subgroup size	10
Learning rate	0.1	Iteration count	300
Expected error	0.01		

**Fig 8:**
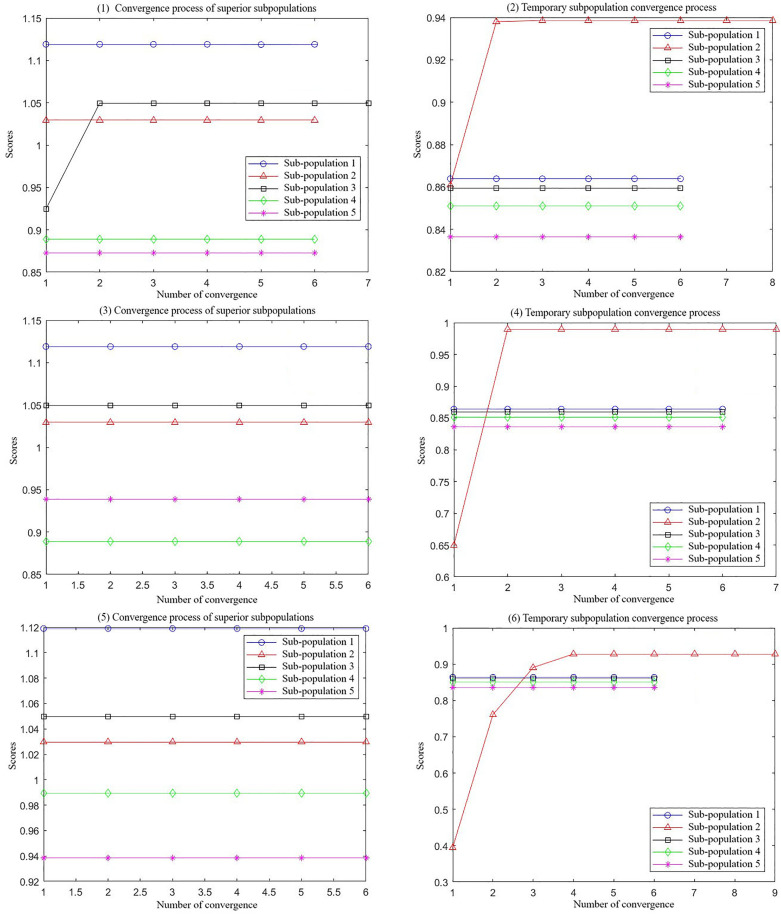
MEA subpopulation convergence processes.

**Fig 9:**
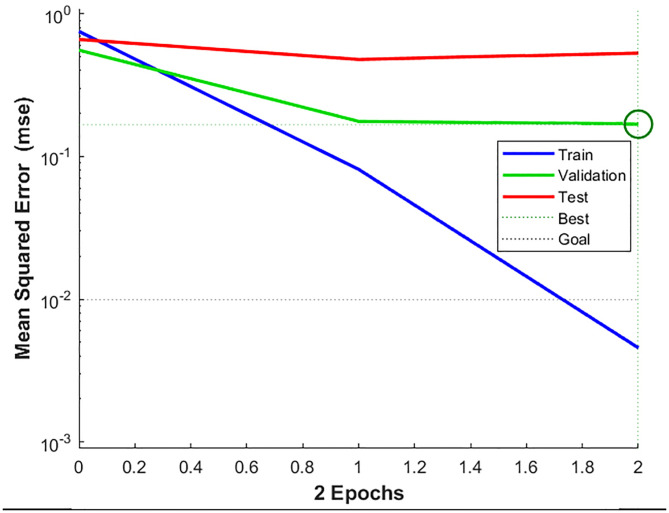
MEA-BP neural network training process curve.

As illustrated in Fig 8, the MEA model undergoes a six-step convergence process. After convergence, all subpopulations reach a mature state. In Fig 8 (1) and (2), elite subpopulations 1, 2, 4, and 5 maintain stable scores without executing convergence operations, as no superior individuals are detected in their vicinity. Meanwhile, temporary subpopulations with lower scores are discarded, and new subpopulations are generated through re-exploration. In Fig 8 (5) and (6), after six iterations of convergence, the highest score among temporary subpopulations falls below the lowest score of elite subpopulations, achieving the global optimal solution. The optimal initial weights and thresholds of the BP neural network are obtained by decoding the best-performing individual and are subsequently used for training. The MSE curve of the MEA-BP prediction model is depicted in Fig 9, demonstrating that the MEA-BP neural network achieves the optimal MSE of 0.16833 within two iterations. Since the final solution does not consist solely of ideal values of 0 or 1, a predefined value range is used as the adoption criterion [41]. Specifically, values within the range of [−0.3, 0.3] are considered as “not adopted”, while those within [0.7, 1.3] are regarded as valid feature values for decision-making. If a value falls outside these ranges, retraining is conducted. The final results are presented in Table 11.

**Table 11:** Decision attribute values for the target case emergency response plan.

Index	Predicted value	Decision value	Index	Predicted value	Decision value
*S*_1_	1	1	*S*_10_	0.1133	0
*S*_2_	1	1	*S*_11_	−0.0188	0
*S*_3_	0.7179	1	*S*_12_	0.9230	1
*S*_4_	1.0318	1	*S*_13_	1	1
*S*_5_	1	1	*S*_14_	1	1
*S*_6_	0.2190	0	*S*_15_	0.8244	1
*S*_7_	1.0912	1	*S*_16_	1	1
*S*_8_	0.8171	1	*S*_17_	1	1
*S*_9_	0.2555	0			

The retrieved similar cases were used to predict the decision values for the target case *C*_0_ using the MEA-BP neural network. Additionally, 50 gas explosion accident cases were selected from the China Coal Mine Safety Production database. Cases with similarity scores below 0.7 were adjusted, and based on the similarity ranking and success of past decisions, an emergency response plan for the target case *C*_0_ was formulated, as detailed in Table 12.

**Table 12:** Emergency response plan for coal mine gas explosion accidents.

	Decision attribute	Specific actions
Emergency Activation	Emergency Rescue Command Headquarters	The emergency response plan is immediately activated, and rescue teams promptly arrive at the scene. A rescue command headquarters is established to coordinate and direct all emergency operations effectively.
	Comprehensive Coordination	
Rescue Actions	Establishment of a Rescue Base	After the gas explosion accident occurred, the gas concentration exceeded the 4% limit. The monitoring personnel immediately reported the situation to the dispatcher, who contacted the underground team by phone to confirm the incident. The dispatcher then issued an evacuation order. The mine director, safety director, chief engineer, and deputy production director proceeded to the mine dispatch center to implement a power shutdown and facilitate evacuation. They then led the mine’s part-time rescue team underground to initiate the rescue operation. Upon receiving the alarm, the mine director, deputy township head, and the head of the safety supervision station arrived at the dispatch center, went to the scene, and entered the mine to assess the situation. If the safety director and other personnel determined that the gas levels were too high, entry would be prohibited. Search operations were conducted along transport tunnels, working faces, and return airways. Victims and injured personnel were located and brought to the surface. Following this, additional search efforts were carried out in other underground tunnels. The deputy township head, head of the safety supervision station, and on-site mine personnel ascended to the surface. The mine’s general manager reported the incident to higher authorities. Rescue teams from county-level and above entered the mine for reconnaissance and joined in the rescue operations. The rescue team entered the working face renovation tunnel to search and identify casualties. The rescue operations concluded once the last victim was recovered from the mine, and the total number of casualties was reported.
	On-Site Monitoring	
	Disaster Area Reconnaissance	
	Direct Fire Suppression	
	Sealed Fire Suppression	
	Search and Rescue of Trapped Personnel	
Emergency Recovery	On-Site Clearance	Immediately after the accident occurs, efforts are initiated for rescue operations and post-accident handling. Relevant department heads must promptly arrive at the site to convey important instructions from higher authorities. They will guide and organize the rescue operations and post-accident handling, ensuring the smooth execution of the rescue, recovery, and investigation processes. Preventive Measures: (1) Implement safety education and practical training activities for coal mines. (2) Thoroughly learn from previous gas explosion accidents in coal mines. (3) Conduct comprehensive rectification of mine safety systems and practices. (4) Launch targeted actions to combat illegal mining operations. (5) Perform a thorough inspection of intermediary organizations involved in coal mine operations. (6) Strengthen the management of on-site safety supervision personnel.
	Medical Treatment	
	Public Communication and Media Guidance	
	Logistical Support	
	Post-Disaster Recovery and Aftercare	

#### 4.1.4 Emergency plan evaluatio.

Eight experts were invited to score the weights of various evaluation indicators. The scoring method and cloud model were used to determine the weight values of each evaluation indicator in the emergency plan, as shown in Table 13.

**Table 13:** Cloud numerical characteristics and weights of each evaluation indicator.

Indicator	*E*_*x*_	*E*_*n*_	*H*_*e*_	Weight	Normalized weight
*R*_1_	0.6337	0.0423	0.0163	0.6337	0.0962
*R*_2_	0.6463	0.0466	0.0192	0.6463	0.0982
*R*_3_	0.5238	0.0411	0.0276	0.5238	0.0796
*R*_4_	0.6962	0.0466	0.0130	0.6962	0.1057
*R*_5_	0.4050	0.0501	0.0161	0.4050	0.0615
*R*_6_	0.4488	0.0544	0.0103	0.4488	0.0682
*R*_7_	0.4100	0.0282	0.0109	0.4100	0.0623
*R*_8_	0.7063	0.0266	0.0078	0.7063	0.1073
*R*_9_	0.6938	0.0298	0.0074	0.6938	0.1054
*R*_10_	0.7088	0.0270	0.0073	0.7088	0.1077
*R*_11_	0.7113	0.0298	0.0136	0.7113	0.1079

Considering the expert evaluations and the actual performance of the target case *C*_0_ emergency plan, the evaluation values from the eight experts are input into the cloud model to obtain the cloud model evaluation values for each indicator, as shown in Table 14.

**Table 14:** Cloud model evaluation values for each indicator.

Indicator	*E*_*x*_	*E*_*n*_	*H*_*e*_	*C*_0_ cloud model evaluation value
*R*_1_	85.83	5.55	1.70	85.83
*R*_2_	77.05	5.38	1.03	77.05
*R*_3_	80.70	6.57	0.81	80.70
*R*_4_	78.76	7.13	0.74	78.76
*R*_5_	81.62	4.11	1.69	81.62
*R*_6_	83.24	6.65	0.70	83.24
*R*_7_	86.81	5.21	2.46	86.81
*R*_8_	87.36	6.87	0.24	87.36
*R*_9_	82.86	9.73	1.70	82.86
*R*_10_	85.54	8.80	1.66	85.54
*R*_11_	81.41	8.35	0.76	81.41

**Fig 10:**
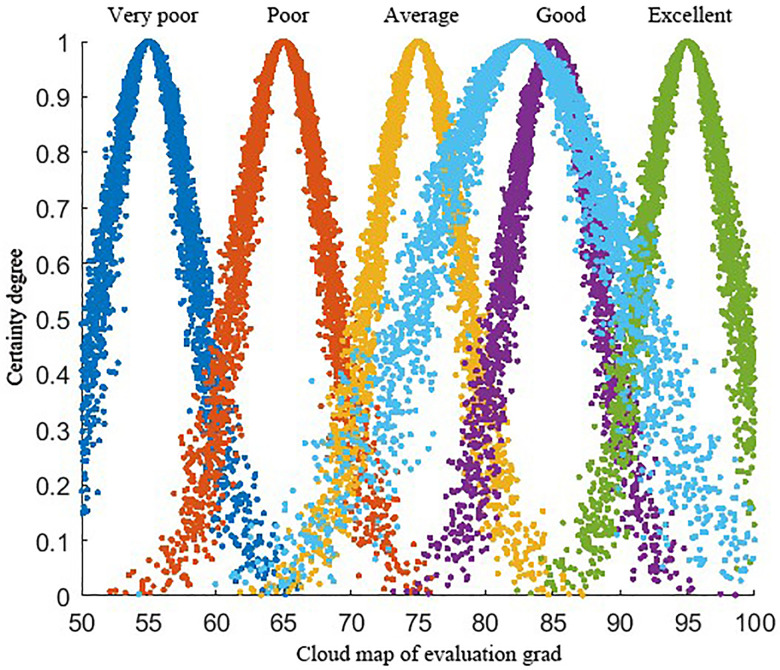
Cloud map of evaluation grad and comprehensive assessment.

The weighted calculation of the indicator weights from Table 13 and the indicator scores from Table 14 results in the comprehensive evaluation cloud for case *C*_0_ emergency plan, *N*(82.78, 7.16, 1.18). This places the evaluation between “Good” and “Fair”, leaning toward a “Good” grade. A comprehensive evaluation cloud map is generated, as shown in Fig 10.

The scores for “Rescue Agency Responsibilities” and “On-site Disposal” were lower, at 77.05 and 78.76, respectively. The remaining scores fall between 80 and 90. The main reasons for the lower scores are the sudden nature of coal mine gas explosion accidents and the significant impact of human factors. The execution ability still requires improvement, and full implementation of intelligent monitoring will require additional time.

### 4.2 Emergency response measures for gas explosion accidents in B coal mine

#### (1) Transportation support.

B Coal Mine must designate specific vehicles for the transportation of essential materials during an accident to ensure their availability. Emergency rescue material routes within the production area must remain unobstructed. For complex mountainous roads, maximum efforts should be made to ensure convenient transportation and effective command. The placement of objects that may obstruct passage in fire lanes is strictly prohibited to prevent traffic congestion during rescue operations.

#### (2) Technical support.

Advanced technological equipment should be adopted to enhance monitoring and surveillance systems. In the event of an accident, early warnings should be issued promptly, and accident data should be collected to predict its progression, ensuring the effective implementation of emergency plans and reducing the risk of escalation. B Coal Mine must be equipped with experts or engineering technicians in production, equipment, technology, safety, and instrumentation, enabling them to provide critical technical information and guidance for decision-making during emergencies.

#### (3) Medical and rescue support.

B Coal Mine’s affiliated hospital should maintain a well-equipped medical team and ample medical supplies. Depending on the development of the situation, arrangements should be made for additional support from higher-level hospitals to ensure timely reinforcement of rescue teams and the fulfillment of emergency medical requirements. The affiliated hospital is responsible for ensuring the availability of emergency rescue medications.

#### (4) Emergency rescue team support.

The internal rescue capabilities of B Coal Mine must be ensured, and relevant functional departments should be mobilized to participate in emergency response actions. In the event of a production safety accident, emergency personnel from each functional department must be assembled by their designated team leaders to form an emergency response team, which should remain stable. Any personnel vacancies due to work adjustments must be promptly filled to ensure adequate rescue forces.

#### (5) Emergency supplies support.

Emergency materials and equipment should be adequately prepared, regularly inspected, and replaced as necessary. Upgrading emergency equipment should be prioritized. Based on the requirements of the emergency response plan, a comprehensive emergency material supply system should be established, with production units as the primary suppliers and social rescue resources as supplementary support. A regional coordination mechanism for emergency material reserves should be improved to enable resource sharing and dynamic management of emergency supplies in B Coal Mine.

## 5. Conclusion

This study addresses the issues in formulating the emergency response plan for gas explosion accidents in B Coal Mine. By employing field investigation, the MEA-BP network, and cloud models, CBR technology was introduced to develop a systematic and structured CBR model. This model enhances the formulation of emergency response plans, improving efficiency, reliability, and effectiveness. The key research findings and conclusions are as follows:

(1) Analysis of the current state and issues in emergency response planning for gas explosion accidents in B Coal Mine. A comprehensive investigation was conducted to examine the current status and challenges in formulating emergency response plans. Identified issues include delays in reporting incidents and establishing emergency command centers, lack of rescue experience, prolonged decision-making times, absence of reference measures, and inability to adjust emergency plans in a timely manner.
(2) Development of a CBR model for formulating emergency response plans for gas explosion accidents. First, characteristics of gas explosion accident cases—including problem descriptions, solutions, and implementation effects—were extracted and standardized. Case representation was conducted using a framework representation method. Based on cloud model theory, a weight calculation method for fundamental attributes was proposed, and an improved KNN algorithm was used for similar case retrieval. The MEA algorithm was employed to optimize the weights and thresholds of BP neural networks, facilitating case reuse. Additionally, the cloud model was incorporated for emergency response plan evaluation and classification.
(3) Simulation of emergency response planning for gas explosion accidents in B Coal Mine. Based on the above research, simulation tests were conducted to validate the reliability of the model. By using the B Coal Mine gas explosion accident as a target case and referencing historical accident cases, a case database was constructed. The CBR model was applied to preliminarily formulate an emergency response plan. The evaluation results indicate that the plan falls between “good” and “average”, leaning towards the “good” category, thereby providing a reference for emergency response planning.
(4) Proposal of implementation and support measures for the emergency response plan. Based on the simulation results, further analyses were conducted on the selection and reuse of emergency plans. Corresponding implementation and support measures were proposed to enhance the effectiveness of the emergency response strategy.

## 6. Outlook

This study integrates Case-Based Reasoning (CBR) into the formulation of emergency response plans for coal mine gas explosions, proposing a novel, structured framework through refinement of key CBR components. Preliminary results demonstrate its potential, yet several limitations—primarily stemming from temporal and technical constraints—remain to be addressed.

(1) While the proposed CBR model provides a systematic foundation for emergency planning, it lacks mechanisms to model inter-agency coordination. Future work should incorporate dynamic, multi-agent collaboration and real-time adaptive strategies to enhance operational relevance and decision-making agility.
(2) The current model has not yet been embedded into a fully functional, integrated emergency response system. Development of user-centric interfaces and seamless interaction with existing coal mine emergency information platforms is critical to support intuitive human–machine collaboration and advance automation in crisis management.
(3) The case dataset employed lacks comprehensive characterization in terms of decision categories, event typologies, and complexity levels. Moreover, no statistical or visual analysis of attribute distributions is currently provided. Future research will address these gaps through descriptive analytics, expanded datasets including international cases, and the identification of potential biases—thereby improving data diversity, representativeness, and robustness.
(4) In light of the inherent uncertainty in real-world scenarios, sensitivity analysis is essential for evaluating model resilience. Although this study focused on model design and initial feasibility, subsequent efforts will quantify how input perturbations influence case retrieval and response outcomes, enhancing both transparency and reliability.

Collectively, future work will prioritize the enrichment of case data, refinement of model granularity, integration with operational systems, and comprehensive stability validation. These advances will lay the groundwork for intelligent, adaptive emergency planning and contribute to improved safety governance in high-risk mining environments.

## Supporting information

S1 File Statistics on gas explosion accidents in China’s coal mines (2000–2025). Basic information and web links of the 50 gas explosion accident cases selected in this study. S2 File. Feature values of basic attributes of accident cases. Characteristic values of the feature attributes for the 50 gas explosion accident cases, calculated according to the method presented in Table 2. S3 File. Characteristic values of decision attributes in accident case emergency response plans. Characteristic values of the decision attributes for gas explosion accident cases with emergency response plans, calculated based on Table 3.
(rar)
